# Clinical management, antifungal susceptibility, and predictors of 28-day mortality in *Candidozyma auris* candidemia: A single-center cohort study

**DOI:** 10.1093/mmy/myag105

**Published:** 2026-09-25

**Authors:** Ayse Kaya, Hatice Rahmet Guner, Imran Hasanoglu, Fatma Eser, Aziz Bozkurt, Yesim Aybar Bilir, Muge Ayhan, Bedia Dinc, Bircan Kayaaslan

**Affiliations:** Department of Infectious Diseases and Clinical Microbiology, Ankara Yıldırım Beyazıt University Faculty of Medicine, Ankara, Türkiye; Department of Infectious Diseases and Clinical Microbiology, Ankara Bilkent City Hospital, Ankara, Türkiye; Department of Infectious Diseases and Clinical Microbiology, Ankara Yıldırım Beyazıt University Faculty of Medicine, Ankara, Türkiye; Department of Infectious Diseases and Clinical Microbiology, Ankara Bilkent City Hospital, Ankara, Türkiye; Department of Infectious Diseases and Clinical Microbiology, Ankara Yıldırım Beyazıt University Faculty of Medicine, Ankara, Türkiye; Department of Infectious Diseases and Clinical Microbiology, Ankara Bilkent City Hospital, Ankara, Türkiye; Department of Infectious Diseases and Clinical Microbiology, Ankara Yıldırım Beyazıt University Faculty of Medicine, Ankara, Türkiye; Department of Infectious Diseases and Clinical Microbiology, Ankara Bilkent City Hospital, Ankara, Türkiye; Department of Infectious Diseases and Clinical Microbiology, Ankara Bilkent City Hospital, Ankara, Türkiye; Department of Infectious Diseases and Clinical Microbiology, Ankara Bilkent City Hospital, Ankara, Türkiye; Department of Infectious Diseases and Clinical Microbiology, Ankara Yıldırım Beyazıt University Faculty of Medicine, Ankara, Türkiye; Department of Infectious Diseases and Clinical Microbiology, Ankara Bilkent City Hospital, Ankara, Türkiye; Department of Medical Microbiology, Health Sciences University, Ankara Bilkent City Hospital, Ankara, Türkiye; Department of Infectious Diseases and Clinical Microbiology, Ankara Yıldırım Beyazıt University Faculty of Medicine, Ankara, Türkiye; Department of Infectious Diseases and Clinical Microbiology, Ankara Bilkent City Hospital, Ankara, Türkiye

**Keywords:** *Candida auris*, *Candidozyma auris*, candidemia, mortality, antifungal resistance, risk factors

## Abstract

*Candidozyma auris* (Candida auris) candidemia is a serious healthcare-associated infection with substantial mortality and limited treatment options. We retrospectively evaluated adult patients with *C. auris* candidemia at a tertiary care center between June 2022 and December 2025 to describe clinical management, microbiological outcomes, antifungal susceptibility, and factors associated with 28-day mortality. The descriptive cohort included 108 patients; mortality status was available for 107. Most patients (92.6%) were in intensive care at candidemia onset, and among evaluable patients, 76.1% had prior *C. auris* colonisation detected. Twenty-eight-day mortality was 41.1% (44/107). Non-survivors were older and had higher Charlson Comorbidity Index, Pitt bacteremia, and Sequential Organ Failure Assessment (SOFA) scores. Central venous catheter removal was more frequent among survivors (72.7% vs. 45.0%; *P* = .012), although removal timing and time to initiation of effective antifungal therapy did not differ significantly between groups. In multivariable analysis, Charlson Comorbidity Index (adjusted odds ratio [aOR], 1.22; 95% confidence interval [CI], 1.02–1.44) and SOFA score (aOR, 1.16; 95% CI, 1.05–1.27) were independently associated with 28-day mortality. Resistance rates were 95.1% for fluconazole, 31.2% for amphotericin B, and 1.3% for both anidulafungin and micafungin; multidrug resistance rate was 28.6%, and no isolate met the definition of pandrug resistance. Mortality in *C. auris* candidemia was mainly associated with baseline comorbidity burden and acute organ dysfunction. Local susceptibility surveillance and early recognition of high-risk patients may support treatment and infection-control strategies.

## Introduction

*Candidozyma auris* (formerly *Candida auris*) is an emerging yeast that causes serious healthcare-associated infections, particularly in critically ill patients with prolonged healthcare exposure. Its frequent antifungal resistance, potential for laboratory misidentification, persistence on skin and environmental surfaces, and capacity to cause difficult-to-control outbreaks have made it a major global public health threat.^1^

*Candidozyma auris* candidemia is associated with mortality rates of approximately 30%–70%, depending on the outcome definition and follow-up period.^2,3^ Disease severity, comorbidity burden, delayed appropriate antifungal therapy, persistent candidemia, and inadequate source control have been associated with adverse outcomes.^4–7^ Geographic variation in antifungal resistance and multidrug resistance further complicates treatment.^8^ Although evidence is increasing, real-world studies integrating treatment, catheter management, microbiological clearance, susceptibility profiles, and short-term mortality remain limited.

This study aimed to evaluate the demographic and clinical characteristics, antifungal treatment, catheter management, microbiological outcomes, and short-term mortality of patients diagnosed with *C. auris* candidemia at our hospital. We also determined the antifungal susceptibility profiles of *C. auris* isolates and identified factors independently associated with 28-day mortality. These findings are expected to contribute to a better understanding of the clinical management of *C. auris* candidemia and to support the development of local treatment and surveillance strategies.

## Materials and methods

### Study aim, design, and setting

This retrospective, single-center observational cohort included adults aged ≥ 18 years with at least one blood culture positive for *C. auris* between June 2022 and December 2025. Only the first candidemia episode per patient was evaluated. Patients with missing data were retained, and analyses used available data for each variable. The primary outcome was 28-day mortality and its associated factors. Secondary outcomes were epidemiological and clinical characteristics, predisposing factors and healthcare exposures, laboratory findings, candidemia complications, and antifungal susceptibility.

The study was approved by the Ankara City Hospital Ethical Committee (TABED 1-24-703).

### Description of materials

Data were retrospectively collected from electronic medical records. Patient age, sex, comorbidities, Charlson Comorbidity Index, and functional status at hospital admission were recorded. For patients admitted to the intensive care unit (ICU), the Acute Physiology and Chronic Health Evaluation II (APACHE II) score was recorded on the day of ICU admission.

To describe the distribution of potential predisposing factors and clinical exposures previously reported in the literature in association with *C. auris* candidemia, the following variables were recorded: hospital unit at candidemia onset, hospitalization and ICU admission within the preceding three months, *C. auris* colonization, multisite colonization, Candida colonization index (CCI), Candida score, steroid or immunomodulatory therapy, chemotherapy, radiotherapy, neutropenia, previous bacteremia and candidemia, and prior antibiotic and antifungal exposure within the month preceding candidemia onset. Devices and supportive interventions present at candidemia onset, including urinary catheters, central venous catheters (CVCs), mechanical ventilation (MV), total parenteral nutrition (TPN), extracorporeal membrane oxygenation, continuous renal replacement therapy, and other indwelling catheters, were also recorded. Device duration was calculated from insertion or initiation to candidemia onset. Other indwelling catheters included arterial and drainage catheters, nephrostomy and feeding tubes, tracheostomy cannulas, and external percutaneous transhepatic cholangiography catheters.

To assess clinical severity, the Pitt bacteremia score and Sequential Organ Failure Assessment (SOFA) score were recorded at the onset of candidemia. These scores were calculated using the worst values recorded within ± 24 h of candidemia onset. The colonization-to-candidemia, hospital admission-to-candidemia, and ICU admission-to-candidemia intervals were calculated. The latter two intervals were calculated for the index hospitalization during which candidemia occurred. The presence of concomitant infection at the time of *C. auris* candidemia was also recorded. The time to initiation of effective antifungal therapy and time to culture clearance were calculated. Among patients with a CVC, CVC removal status and the time from candidemia onset to CVC removal were recorded. The antifungal agents administered and the duration of antifungal therapy were also evaluated.

### Definitions

Candidemia onset was defined as the date of collection of the first blood culture positive for *C. auris*. Twenty-eight-day mortality was defined as all-cause mortality occurring within 28 days after the onset of candidemia.

Comorbidities included chronic cardiovascular disease (hypertension, coronary artery disease, heart failure, arrhythmia, or previous valve surgery), chronic neurological disease (Alzheimer’s or Parkinson’s disease or previous cerebrovascular event), diabetes mellitus, chronic kidney disease (GFR <60 ml/min/1.73 m²), dialysis, immunosuppressive conditions (solid organ transplantation, solid or hematologic malignancy, or HIV infection), and other chronic conditions.

Functional status at admission was categorized by ability to perform activities of daily living (ADLs): fully independent; requiring minor assistance (guidance, supervision, or minimal mobility support); requiring major assistance with basic ADLs; or completely dependent (immobilized, bedridden, or dependent on caregivers for daily care).

Previous hospitalization and ICU admission were defined as hospitalization or ICU stay, respectively, within the 3 months preceding *C. auris* candidemia.

Routine colonization screening was not performed for all patients or for all ICU patients. *Candidozyma auris* colonization screening was conducted as part of targeted infection-control surveillance in epidemiologically linked patients, including those sharing healthcare personnel or medical equipment. Pooled bilateral axillary and inguinal swabs were collected in Stuart transport medium. Colonization was defined as isolation of *C. auris* from at least one non-sterile site before candidemia without clinical infection; multisite colonization required more than one site. The Candida colonization index (CCI), calculated only in colonized patients, was the ratio of colonized to cultured sites.^9^ CCI was classified as low (<0.5) or high (≥0.5).

The candida score was calculated according to previously established criteria.^10^

Catheter-related candidemia was defined in patients with a CVC according to the differential time-to-positivity criterion, with the CVC blood culture becoming positive at least 2 h earlier than the simultaneously collected peripheral blood culture.

Concomitant infection was defined as the presence of another infection diagnosed clinically and microbiologically at the time of *C. auris* candidemia.

Effective therapy was defined, when susceptibility results were available, as administration of an antifungal agent to which the isolate was susceptible *in vitro*. Time to effective therapy was calculated from index blood culture collection. Culture clearance was the first negative blood culture after the index culture, provided subsequent cultures remained negative during the episode. Analyses of treatment duration excluded patients whose treatment ended because of death or whose duration data were unavailable.

Delayed CVC removal was defined as removal of the CVC more than 24 h after collection of the index blood culture that subsequently yielded *C. auris*. This analysis was restricted to patients whose CVC was removed and for whom removal timing was available; patients whose CVC was not removed during the candidemia episode or whose removal timing was unavailable were excluded.

Recurrent *C. auris* candidemia was defined as a new episode of *C. auris* bloodstream infection occurring after documented culture clearance and completion of the initial antifungal treatment course.

Complications of *C. auris* candidemia included endophthalmitis, valvular vegetation, and hepatosplenic candidiasis; rates were calculated among patients evaluated for each complication.

### Microbiological analysis

Isolate identification and antifungal susceptibility data were obtained from the laboratory information system and, when applicable, the national public health database. Isolates were identified using MALDI-TOF MS (Bruker, Billerica, USA).

Until September 2023, isolates were referred to the National Reference Laboratory for broth microdilution testing; thereafter, testing was performed locally using the commercial broth microdilution-based Sensititre YeastOne system (Thermo Fisher Scientific, USA).

Testing was unavailable for some isolates because stored strains could not be recovered or were unavailable. Minimal inhibitory concentrations (MICs) were interpreted using tentative Centers for Disease Control and Prevention (CDC) breakpoints.^11^ No tentative CDC breakpoint is available for voriconazole; therefore, voriconazole MIC values were reported descriptively and were not categorized as susceptible or resistant. Multidrug resistance (MDR) and pandrug resistance (PDR) were defined as resistance to at least two and all three, respectively, of the following antifungal categories: azoles, represented by fluconazole; polyenes, represented by amphotericin B; and echinocandins. Resistance to anidulafungin or micafungin was sufficient to classify an isolate as echinocandin-resistant. An isolate was classified as non-resistant to echinocandins only when results were available for both anidulafungin and micafungin and neither agent was categorized as resistant. Susceptibility to only one of these agents, with the other result missing, was considered insufficient to classify the echinocandin category as non-resistant. Caspofungin results were reported descriptively but were not used to determine echinocandin-class resistance or MDR/PDR status because of known intermethod variability and the potential Eagle effect. MDR and PDR were assessed only in isolates for which all three antifungal categories were evaluable. MIC₅₀ and MIC₉₀ were calculated from all available results and represent pooled distributions from the two broth microdilution-based methods.

### Statistical analysis

Analyses were performed in Python using pandas, NumPy, SciPy, statsmodels, and scikit-learn. Continuous variables were summarized as median (interquartile range [IQR]) and categorical variables as number (%).

Patients were compared according to 28-day mortality status. Continuous variables were compared using the Mann–Whitney U test. Categorical variables were compared using the chi-square test or Fisher’s exact test, as appropriate.

Variables associated with mortality in univariable analysis and clinically relevant variables were considered for multivariable logistic regression. The final model was selected according to clinical relevance, statistical significance, and stability to limit overfitting. Results are reported as odds ratios (ORs) with 95% CIs. Pitt and SOFA scores were not entered together because of conceptual overlap; SOFA was selected because it more broadly measures organ dysfunction, while Pitt score was assessed separately by receiving operating characteristic (ROC) analysis. Calibration was evaluated with the Hosmer–Lemeshow test and discrimination with AUC.

ROC analyses evaluated platelet count, C-reactive protein, procalcitonin, D-dimer, Pitt bacteremia score, and *C. auris* blood culture TTP, selected according to clinical relevance and data availability. Laboratory values closest to candidemia onset were used. AUC with 95% confidence interval (CI), Youden index-derived optimal cutoff, sensitivity, specificity, positive predictive value, and negative predictive value were calculated; AUCs were tested against 0.5.

All analyses were performed using available data for each variable, and missing data were not imputed. A two-sided *P* value of < 0.05 was considered statistically significant.

## Results

A total of 108 patients were included in the descriptive cohort. Mortality status was unavailable for one patient; therefore, comparisons according to 28-day mortality were performed in 107 patients, including 44 who died and 63 who survived. The 28-day mortality rate was 41.1% (44/107). Demographic and baseline characteristics and comorbidities are summarized in Table 1. The median age of the patients was 71.5 years (IQR, 55–81), and 64 (59.3%) were male. The median APACHE II score was 19 (IQR, 14–25.2). Patients who died within 28 days were significantly older than survivors (median, 77 years [IQR, 70.5–83] vs 64 years [IQR, 49–77]; *P* < 0.001). No significant differences were observed between non-survivors and survivors in terms of sex or APACHE II score. Regarding comorbidities, 57 patients (52.8%) had three or more comorbid conditions. The most common comorbidities were chronic cardiovascular disease (61.1%), chronic neurological disease (48.1%), diabetes mellitus (35.2%), and chronic kidney disease (26.9%). Chronic cardiovascular disease was more frequent among non-survivors than among survivors (75.0% vs. 50.8%; *P* = 0.020). Charlson Comorbidity Index was also significantly higher in non-survivors (median, 5 [IQR, 5–7.2] vs. 4 [IQR, 3–6]; *P* = 0.009).

**Table 1.** Characteristics of patients with *C. auris* candidemia according to 28-day mortality (all patients, *n* = 108; patients with available 28-day outcome data, *n* = 107).

	All patients *n* = 108 (%)	Non-survivors *n* = 44 (%)	Survivors *n* = 63 (%)	*P* value*	Crude OR (95% CI)**
**Demographics and baseline characteristics**					
Age, years, median (IQR)	71.5 (55–81)	77 (70.5–83)	64 (49–77)	**<0.001**	—
Male sex	64 (59.3)	25/44 (56.8)	39/63 (61.9)	0.743	0.81 (0.37–1.77)
APACHE II score, median (IQR) (*n* = 100, 42, 58)***	19 (14–25.2)	19 (14–25.8)	18.5 (14–24)	0.883	—
**Comorbidities**					
≥3 comorbidities	57 (52.8)	28 (63.6)	29 (46)	0.110	2.05 (0.93–4.52)
Chronic cardiovascular disease	66 (61.1)	33 (75)	32 (50.8)	**0.020**	**2.91 (1.25–6.75)**
Chronic neurological disease	52 (48.1)	26 (59.1)	25 (39.7)	0.075	2.20 (1.00–4.81)
Diabetes mellitus	38 (35.2)	16 (36.4)	22 (34.9)	1.000	1.06 (0.48–2.38)
Chronic kidney disease	29 (26.9)	16 (36.4)	13 (20.6)	0.114	2.20 (0.92–5.22)
Dialysis	26 (24.1)	15 (34.1)	11 (17.5)	0.081	2.45 (0.99–6.02)
Immunosuppressive diseases^****^	22 (20.4)	11 (25.0)	11 (17.5)	0.480	1.58 (0.61–4.04)
Chronic pulmonary disease	21 (19.4)	9 (20.5)	12 (19.0)	1.000	1.09 (0.42–2.87)
Others^*****^	18 (16.7)	7 (15.9)	11 (17.5)	1.000	0.89 (0.32–2.52)
**Charlson Comorbidity Index, median (IQR)**	5 (3–7)	5 (5–7.2)	4 (3–6)	**0.009**	—
Functional status at hospital admission (*n* = 107, 44, 62)^**^					
Completely dependent	45 (42.1)	17 (38.6)	27 (43.5)	0.813	0.82 (0.37–1.79)
Fully independent	38 (35.5)	14 (31.8)	24 (38.7)	0.644	0.74 (0.33–1.67)
Minor assistance with ADLs	19 (17.7)	10 (22.7)	9 (14.5)	0.386	1.73 (0.64–4.70)
Major assistance with ADLs	5 (4.7)	3 (6.8)	2 (3.2)	0.400	2.20 (0.35–13.72)
**Location at candidemia onset**					
Intensive care unit	100 (92.6)	42 (95.5)	58 (92.1)	0.697	1.81 (0.33–9.78)
Ward	7 (6.5)	2 (4.5)	5 (7.9)		0.55 (0.10–2.99)
Emergency department	1 (0.9)	–	–		NE
**Pre-candidemia predisposing factors and exposures**					
Hospitalization before admission	41 (38.0)	19/44 (43.2)	22/63 (34.9)	0.507	1.42 (0.64–3.12)
ICU stay before admission	35 (32.4)	16/44 (36.4)	19/63 (30.2)	0.643	1.32 (0.58–2.99)
*C. auris* colonization	51/67 (76.1)	20/26 (76.9)	31/41 (75.6)	1.000	1.08 (0.34–3.42)
Multisite colonization	14/67 (20.9)	3/26 (11.5)	11/41 (26.8)	0.217	0.36 (0.09–1.42)
Candida colonization index, median (IQR) (n = 51, 31, 20)***	0.3 (0.3-0.6)	0.3 (0.3–0.6)	0.3 (0.3–0.6)	1.000	—
Low Candida colonization index	29/51 (56.9)	11/20 (55.0)	18/31 (58.1)	1.000	0.88 (0.28–2.74)
High Candida colonization index	22/51 (43.1)	9/20 (45.0)	13/31 (41.9)	1.000	1.13 (0.36–3.52)
Candida score, median (IQR)	2 (1-3)	2 (1–3)	2 (1–3)	0.740	—
Steroid/immunomodulator use	26 (24.1)	10 (22.7)	16 (25.4)	0.930	0.86 (0.35–2.14)
Chemotherapy	3 (2.8)	1 (2.3)	2 (3.2)	1.000	0.71 (0.06–8.07)
Radiotherapy	1 (0.9)	0 (0.0)	1 (1.6)	1.000	NE
Neutropenia	1 (0.9)	1 (2.3)	0 (0.0)	0.411	NE
Antibiotic use before candidemia	94 (87.0)	42 (95.5)	52 (82.5)	0.069	4.44 (0.93–21.15)
Antifungal use before candidemia	33 (30.6)	17 (38.6)	16 (25.4)	0.213	1.85 (0.81–4.24)
Previous bacteremia	60/107 (56.1)	24/44 (54.5)	36/62 (58.1)	0.872	0.87 (0.40–1.89)
Previous candidemia	11/107 (10.3)	6/44 (13.6)	5/62 (8.1)	0.546	1.80 (0.51–6.32)
Pressure ulcer	78 (72.2)	32 (72.7)	45 (71.4)	1.000	1.07 (0.45–2.52)
MDR organism colonization and/or infection	90/107 (84.1)	37/44 (84.1)	53/62 (85.5)	1.000	0.90 (0.31–2.63)
**Invasive devices and supportive interventions at candidemia onset**					
Urinary catheter	104 (96.3)	44 (100.0)	60 (95.2)	0.267	NE
Urinary catheter duration, days, median (IQR) (*n* = 102, 44, 59)***	35 (22–52)	29.5 (19.8–39.2)	41.5 (26.5–63.8)	**0.009**	—
Central venous catheter use	95 (88.0)	40 (90.9)	55 (87.3)	0.758	1.45 (0.41–5.17)
CVC duration, days, median (IQR) (*n* = 89, 40, 49)***	23 (15–41)	20 (15–34.2)	28 (18–48)	0.109	—
Mechanical ventilation	74 (68.5)	34 (77.3)	40 (63.5)	0.192	1.96 (0.82–4.68)
MV duration, days, median (IQR) (*n* = 73, 34, 39)***	36 (17–82)	26 (16.2–41)	56 (20.5–92.5)	**0.031**	—
Total parenteral nutrition	14 (13.0)	2 (4.5)	12 (19.0)	**0.040**	0.20 (0.04–0.96)
TPN duration, days, median (IQR) (*n* = 14, 2, 12)***	32.5 (21–43.8)	42 (33–51)	32.5 (18.8-41.2)	0.648	—
Extracorporeal membrane oxygenation	7 (6.5)	4 (9.1)	3 (4.8)	0.442	2.00 (0.42–9.42)
Continuous renal replacement therapy	4 (3.7)	3 (6.8)	1 (1.6)	0.303	4.54 (0.46–45.13)
Other indwelling catheters	63 (58.3)	21 (47.7)	41 (65.1)	0.112	0.49 (0.22–1.08)
**Clinical severity at candidemia onset**					
Pitt bacteremia score, median (IQR)	8 (3–12)	10 (6.8–12)	6 (1–10)	**<0.001**	—
SOFA score, median (IQR)	9 (5–12)	10.5 (7.8–14)	7 (4–10)	**<0.001**	—
Colonization-to-candidemia interval, days, median (IQR) (*n* = 51, 20, 31)***	19 (8–36)	10 (2–26)	24 (13–46)	**0.013**	—
Admission-to-candidemia interval, days, median (IQR)	44.5 (24.8–69.8)	34.5 (22.8–56.8)	52 (32–94.5)	**0.013**	—
ICU admission-to-candidemia interval, days, median (IQR) (*n* = 100, 42, 58)***	37 (21–62)	29 (16–46)	44.5 (23.5–76.5)	**0.030**	—
Time to positivity of *C. auris* blood culture, hours, median (IQR) (*n* = 100, 42, 58)***	26 (20–31)	27 (24–31)	24 (16.5–30)	0.193	—
Catheter-related candidemia	28/95 (29.5)	12/40 (30)	16/55 (29.1)	1.000	1.04 (0.43–2.55)
Concomitant infection	27 (25.0)	9 (20.5)	18 (28.6)	0.468	0.64 (0.26–1.60)
Time to initiation of effective therapy, days, median (IQR) (*n* = 84, 31, 53)***	2 (1–3)	2 (1–3)	2 (1–3)	0.886	—
Time to culture clearance, days, median (IQR) (*n* = 59, 13, 46)***	6 (3.5–10)	3 (2–8)	6 (4–11.8)	0.082	—
CVC removal	58/95 (61.1)	18/40 (45.0)	40/55 (72.7)	**0.012**	**0.31 (0.13–0.73)**
Delayed CVC removal	7/51 (13.7)	3/14 (21.4)	4/37 (10.8)	0.376	2.25 (0.43–11.66)
Total duration of antifungal therapy, days, median (IQR) (*n* = 52, 8, 44)***	19 (15–26)	23.5 (15–32.5)	18 (15–26)	0.541	—
Duration of antifungal therapy after culture clearance, days, median (IQR) (*n* = 49, 6, 43)***	14 (14–16)	15.5 (14–20)	14 (14–15)	0.382	—
Secondary bacteremia during antifungal therapy	40/96 (41.7)	12/34 (35.3)	28/62 (45.2)	0.471	0.66 (0.28–1.57)
Recurrent *C. auris* candidemia	8/61 (13.1)				

IQR, interquartile range; APACHE II, Acute Physiology and Chronic Health Evaluation II; ADL, activities of daily living; ICU, intensive care unit; CVC, central venous catheter; MV, mechanical ventilation, TPN, total parenteral nutrition; SOFA, Sequential Organ Failure Assessment.

* *P* values indicate comparisons between patients who died and those who survived.

** Crude ORs were calculated for categorical variables, with 28-day mortality as the outcome and the absence of the evaluated characteristic as the reference category. ORs were not calculated for continuous variables.

*** When three sample sizes are provided in parentheses, they refer to all patients, non-survivors, and survivors, respectively.

**** Ten patients had undergone solid organ transplantation (SOT), six had hematologic malignancy, seven had solid organ malignancy, and one had HIV infection.

***** Other comorbidities included thyroid disease (*n* = 8; hypothyroidism, *n* = 7; hyperthyroidism, *n* = 1), benign prostatic hyperplasia (*n* = 6), chronic rheumatologic disease (*n* = 3), chronic liver disease (*n* = 2), chronic hepatitis B (*n* = 1), and Down syndrome (*n* = 1).

There was no significant difference between the groups in the distribution of functional status at hospital admission (Table 1). Of the patients, 45 (42.1%) were completely dependent and 38 (35.5%) were fully independent. At the onset of candidemia, the vast majority of patients were being treated in the ICU (100/108, 92.6%).

Pre-candidemia predisposing factors and exposures are summarized in Table 1. Pre-candidemia, 41 patients (38.0%) had hospitalization before admission and 35 (32.4%) had ICU stay before admission. Among evaluable patients, *C. auris* colonization was detected in 76.1%, and multisite colonization in 20.9%. The CCI and Candida score did not differ significantly between non-survivors and survivors (Table 1). Antibiotic use before candidemia was documented in 94 patients (87.0%) and 33 patients (30.6%) had received antifungal therapy. Class-specific antifungal exposure data were available for 106 patients: 21 (19.8%) had received an echinocandin, 14 (13.2%) an azole, and 4 (3.8%) amphotericin B. Some patients had received more than one antifungal class; therefore, these categories were not mutually exclusive. Antibiotic and antifungal use before candidemia was similar between non-survivors and survivors. The most frequently used antibiotic classes were carbapenems (75.9%), glycopeptides or linezolid (62.0%), beta-lactams (59.3%), and polymyxins (48.1%). Previous bacteremia and previous candidemia were documented in 56.1% and 10.3% of patients, respectively. No statistically significant differences were observed between non-survivors and survivors for these variables (all *P* > 0.05).

The frequencies of invasive devices and supportive interventions present at candidemia onset and their comparisons between groups are presented in Table 1. The most common were urinary catheterization (96.3%), CVC use (88.0%), mechanical ventilation (MV; 68.5%), and total parenteral nutrition (TPN; 13.0%). Except for TPN use, which was less frequent among non-survivors than survivors (4.5% vs. 19.0%; *P* = 0.040), no statistically significant differences were observed between the groups for these variables (all *P* > 0.05). However, the median durations of urinary catheterization and mechanical ventilation before candidemia onset were significantly longer among survivors than non-survivors (urinary catheterization: 41.5 (26.5–63.8) vs. 29.5 (19.8–39.2) days; *P* = 0.009; mechanical ventilation: 56 (20.5–92.5) vs. 26 (16.2–41) days; *P* = 0.031).

In the comparison of clinical severity at candidemia onset, the median Pitt bacteremia and SOFA scores were significantly higher among non-survivors than survivors (10 (6.8–12) vs. 6 (1–10), *P* < 0.001; and 10.5 (7.8–14) vs. 7 (4–10), *P* < 0.001, respectively).

The colonization-to-candidemia, hospital admission-to-candidemia, and ICU admission-to-candidemia intervals were significantly shorter among non-survivors than survivors (Table 1). The median time to positivity of *C. auris* blood cultures was 26 h (20–31) and did not differ significantly between non-survivors and survivors (27 [24–31] vs. 24 [16.5–30] hours; *P* = 0.193). Catheter-related candidemia was identified in 28 patients, corresponding to 25.9% of the overall study cohort and 29.5% of the 95 patients with a CVC, and was the only microbiologically confirmed source. The source could not be reliably determined for the remaining episodes. Possible concurrent extra-bloodstream *C. auris* infection was recorded in six patients; however, the anatomical sites and supporting diagnostic details were unavailable, precluding classification as confirmed extra-bloodstream infection. Concomitant infection was present in 27 patients (25.0%). Catheter-related candidemia and concomitant infection were comparable between non-survivors and survivors (12/40 [30.0%] vs. 16/55 [29.1%], *P* = 1.000; and 9/44 [20.5%] vs. 18/63 [28.6%], *P* = 0.468, respectively). All concomitant infections were bacterial. Of these, 24 were ventilator-associated pneumonia, two were urinary tract infections, and one was a catheter-related bloodstream infection. The median time to initiation of effective therapy was 2 days (1–3) and was similar between non-survivors and survivors (2 [1–3] vs. 2 [1–3] days; *P* = 0.886). The median time to culture clearance was shorter among non-survivors than survivors (3 [2–8] vs. 6 [4–11.8] days), although the difference was not statistically significant (*P* = 0.082). Among the 95 patients with a CVC, the catheter was removed in 58 (61.1%). The CVC removal rate was significantly lower among non-survivors than survivors (18/40 [45.0%] vs. 40/55 [72.7%]; *P* = 0.012). Among the 58 patients whose CVC was removed, removal timing was available for 51. The rate of delayed CVC removal was similar between non-survivors and survivors (3/14 [21.4%] vs. 4/37 [10.8%]; *P* = 0.376).

The total duration of antifungal therapy was calculated after excluding patients whose treatment was discontinued because of death. The median total duration of antifungal therapy was 19 days (15–26), and the median duration of antifungal therapy after culture clearance was 14 days (14–16). Antifungal treatment durations were similar between non-survivors and survivors (*P* = 0.541 for total treatment duration and *P* = 0.382 for treatment duration after culture clearance). Anidulafungin and micafungin were each initiated in 41 of 96 patients (42.7%), caspofungin in 7 (7.3%), fluconazole in 4 (4.2%), and amphotericin B in 3 (3.1%). Twelve patients died before receiving antifungal therapy. Secondary bacteremia during antifungal therapy was detected in 40 of 96 patients (41.7%), whereas recurrent *C. auris* candidemia was identified in 8 of 61 evaluable patients (13.1%). Recurrent *C. auris* candidemia occurred in 1 of 2 non-survivors (50.0%) and in 7 of 59 survivors (11.9%). Because recurrent *C. auris* candidemia could only be assessed in patients who survived long enough to achieve culture clearance and complete the initial treatment course, no comparison according to 28-day mortality was performed.

In the final multivariable logistic regression model, 107 patients with complete data for the selected variables were included, with 44 deaths within 28 days. The candidate variables were older age, Charlson Comorbidity Index, TPN use, durations of MV and urinary catheterization, Pitt bacteremia and SOFA scores, the intervals from colonization, hospital admission, and ICU admission to candidemia onset, and CVC removal. Clinical relevance, the extent of missing data, variable distributions, conceptual overlap, and model stability were considered together during model development. Age was not entered together with the age-adjusted Charlson Comorbidity Index, and Pitt bacteremia and SOFA scores were not entered simultaneously because of conceptual overlap. Variables available only in selected subgroups or producing unstable estimates were not retained. The final baseline model included the Charlson Comorbidity Index and SOFA score. In the final model, Charlson Comorbidity Index (adjusted OR, 1.22; 95% CI, 1.02–1.44; *P* = 0.025) and SOFA score (adjusted OR, 1.16; 95% CI, 1.05–1.27; *P* = 0.003) were independently associated with 28-day mortality. CVC removal was associated with survival in univariable analysis; however, it was not included in the primary baseline model because it was a post-baseline intervention available only in patients with a CVC and was susceptible to early-mortality bias. The final model was therefore restricted to baseline clinical variables. The final model demonstrated acceptable discrimination with an AUC of 0.728 and good calibration by the Hosmer–Lemeshow test (*P* = 0.947).

The discriminatory performance of clinical scores and laboratory parameters for predicting 28-day mortality was evaluated using ROC analysis (Table 2 and Fig. 1). For the Pitt bacteremia score, the AUC was 0.690 (95% CI, 0.590–0.790; *P* < 0.001), and the optimal cutoff value was ≥ 12. For procalcitonin, the AUC was 0.707 (95% CI, 0.603–0.811; *P* < 0.001), with an optimal cutoff value of ≥ 0.5 µg/l. For CRP, the AUC was 0.633 (95% CI, 0.524–0.741; *P* = 0.017), and the optimal cutoff value was ≥204 mg/l. For D-dimer, the AUC was 0.659 (95% CI, 0.539–0.778; *P* = 0.009), with an optimal cutoff value of ≥ 3.13 mg/l. For platelet count, the AUC was 0.712 (95% CI, 0.614–0.811; *P* < 0.001), and the optimal cutoff value was ≤ 208 × 10⁹/l. TTP showed no discriminatory ability for 28-day mortality (AUC, 0.424; *P* = 0.182).

**Figure 1.**
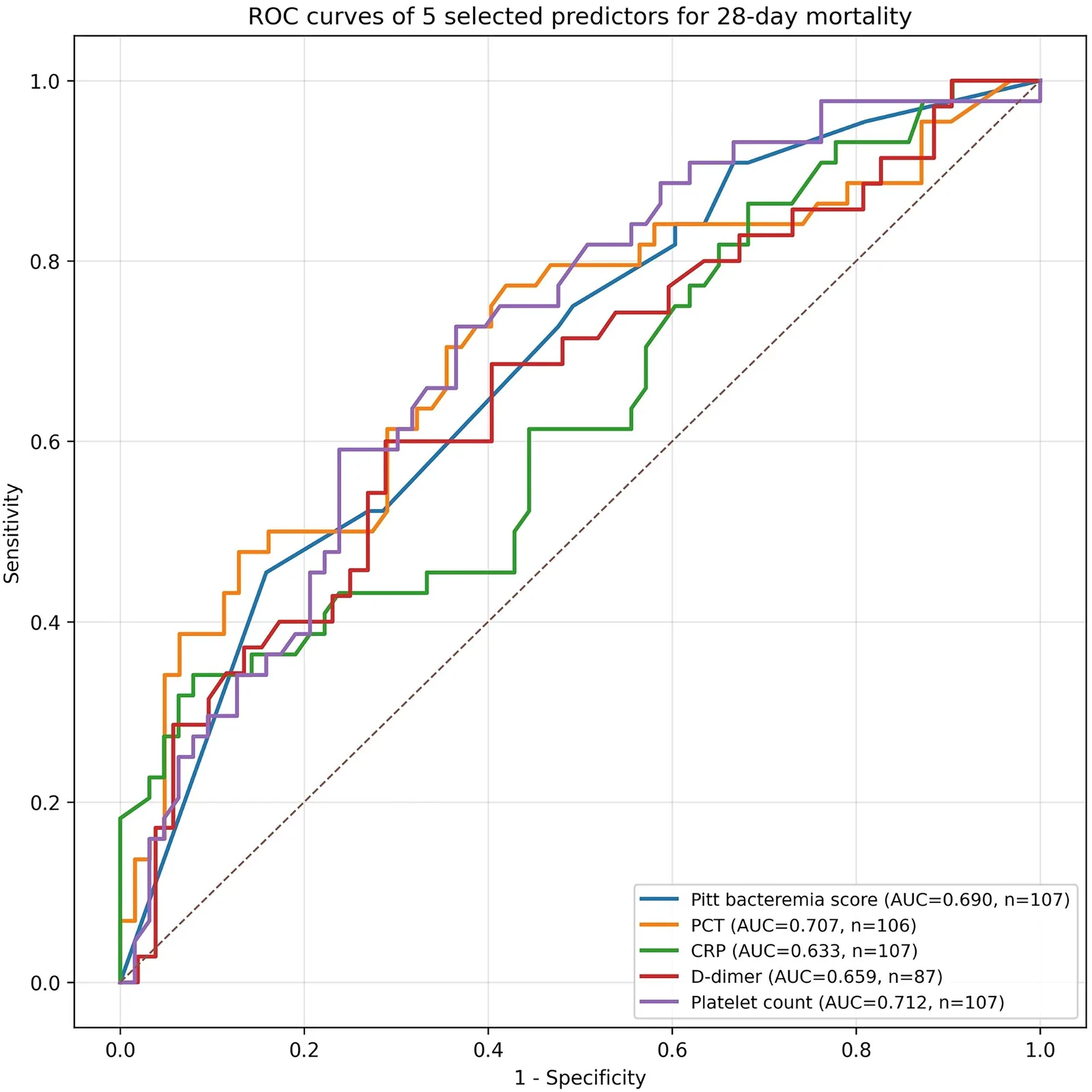
Receiver operating characteristic curves of selected clinical and laboratory predictors for 28-day mortality in patients with *C. auris* candidemia. PCT, procalcitonin; CRP, C-reactive protein.

**Table 2.** Predictive performance of Pitt bacteremia score, laboratory parameters and time to positivity of *C. auris* blood culture for 28-day mortality.

Parameter	AUC (95% CI)	*P* value	Optimal cutoff	Sensitivity (%)	Specificity (%)	PPV (%)	NPV (%)
Platelet count (*n* = 107)	0.712 (0.614–0.811)	<0.001	≤208 × 10⁹/l	72.7	63.5	58.2	76.9
Procalcitonin (*n* = 106)	0.707 (0.603–0.811)	<0.001	≥0.5 µg/l	77.3	58.1	56.7	78.3
Pitt bacteremia score (*n* = 107)	0.690 (0.590–0.790)	<0.001	≥12	45.5	84.1	66.7	68.8
D-dimer (*n* = 87)	0.659 (0.539–0.778)	0.009	≥3.13 mg/l	60.0	71.2	58.3	72.5
CRP (*n* = 107)	0.633 (0.524–0.741)	0.017	≥204 mg/l	34.1	92.1	75.0	66.7
Time to positivity of *C. auris* blood culture (*n* = 100)	0.424 (0.313–0.536)	0.182	—	—	—	—	—

AUC, area under the receiver operating characteristic curve; CI, confidence interval; CRP, C-reactive protein; NPV, negative predictive value; PPV, positive predictive value.

As part of the evaluation for complications, fundoscopic examination was performed in 52 patients (48.1%), and endophthalmitis was detected in 1 of the evaluated patients (1.9%). Echocardiography was performed in 56 patients (51.9%), and valvular vegetation was identified in 2 (3.6%). Abdominal imaging was performed in 40 patients(37.0%), and no cases of hepatosplenic candidiasis were detected (Table 3).

**Table 3.** *Candidozyma auris* candidemia complications and antifungal resistance profile.

	n/N (%)		
Echocardiography performed	56/108 (51.9)		
Vegetation among patients undergoing echocardiography	2/56 (3.6)		
Fundoscopic examination performed	52/108 (48.1)		
Endophthalmitis among examined patients	1/52 (1.9)		
Abdominal imaging performed	40/108 (37.0)		
Hepatosplenic candidiasis among imaged patients	0/40 (0.0)		
Antifungal resistance	n (resistant)/N (tested) (%)	MIC₅₀ (µg/ml)	MIC₉₀ (µg/ml)
Fluconazole resistance	77/81 (95.1)	256	>256
Voriconazole (descriptive MICs only)	—	0.5	2
Amphotericin B resistance	25/80 (31.2)	1	2
Caspofungin MIC ≥2 µg/ml ^†^	7/82 (8.5)	0.25	1
Anidulafungin resistance	1/79 (1.3)	0.12	0.25
Micafungin resistance	1/80 (1.3)	0.12	0.25
MDR*	22/77 (28.6)		
PDR*	0/77 (0.0)		

MDR, multidrug resistance; PDR, pandrug resistance.

† Caspofungin results were reported descriptively and were not used to determine echinocandin-class resistance or MDR/PDR status because of known intermethod variability and the potential Eagle effect.

* MDR and PDR classifications were based on the study definitions provided in the Methods and may not be directly comparable with studies using different drug panels or category definitions.

Among isolates with available antifungal resistance results, resistance was detected in 77 of 81 isolates (95.1%) for fluconazole, 25 of 80 (31.2%) for amphotericin B, 1 of 79 (1.3%) for anidulafungin, and 1 of 80 (1.3%) for micafungin. Seven of 82 isolates (8.5%) had a caspofungin MIC at or above the CDC tentative threshold; these results were reported descriptively but were not used to determine echinocandin-class resistance or MDR/PDR status. Because no tentative CDC interpretive breakpoint is available for voriconazole, voriconazole MIC values were reported descriptively. Among the 77 isolates for which all three antifungal categories were evaluable, 22 (28.6%) met the definition of MDR, whereas no isolate met the revised definition of PDR (0/77). Consequently, an association between PDR and 28-day mortality and PDR-specific alternative treatment patterns could not be evaluated (Table 3).

## Discussion

This 3.5-year cohort provides an integrated evaluation of *C. auris* candidemia, including clinical management and antifungal resistance. Most infections occurred in critically ill patients with invasive devices, and colonization was common.

Twenty-eight-day mortality was 41.1%, within the reported range of 30.1%–71.4%.^12–16^ Variation likely reflects differences in outcome definitions, case mix, clinical severity, and candidemia management; a 2009–2025 review estimated overall mortality at approximately 30%, with substantial geographic and patient-level variation.^6^

Among 187 patients with *C. auris* candidemia, in-hospital mortality was 54.6%, culture clearance occurred in 66.9% of those with follow-up cultures, and Pitt score, hemodialysis, and hospital stay were associated with mortality.^4^ In the international prospective study by Erdem et al., 30-day mortality was 56.2%; ICU admission, abdominal surgery, thrombocytopenia, inadequate access to appropriate antifungals, CVC presence, and deep-seated complications were associated with adverse outcomes.^17^

In our study, Charlson Comorbidity Index and SOFA score were independently associated with mortality. Platelet count and procalcitonin showed the highest ROC discrimination (AUC, 0.712 and 0.707); Pitt score, D-dimer, and CRP were also informative, whereas TTP was not. These findings suggest that baseline comorbidity and acute organ dysfunction were the main mortality determinants. A Turkish ICU study similarly associated SOFA and older age with mortality, while other reports identified multiple catheters, recent ICU admission, and intubation.^7,18^ Differences across studies may reflect case mix, severity, treatment access, and local management.

CVC removal was more frequent among survivors, but patients who died may not have survived long enough for removal, introducing survivor bias. Among patients whose CVC was removed and for whom removal timing was available, delayed removal was approximately twice as frequent among non-survivors as among survivors (21.4% vs. 10.8%). Although this difference was not statistically significant, its direction may be clinically relevant and consistent with a potential benefit of prompt source control. The relatively low proportion of delayed CVC removal, despite a median time to blood culture positivity of approximately 26 h, may reflect the prompt empirical removal of temporary CVCs as an early source-control measure in critically ill or septic patients, without awaiting blood culture positivity. However, the analysis included only 51 patients and seven delayed-removal events and excluded patients whose CVC was never removed, resulting in limited statistical power and susceptibility to selection, survivor, and early-mortality biases. Time to effective antifungal therapy did not differ according to survival status. Therefore, our findings cannot establish a causal relationship between CVC removal timing and mortality. Although CVC presence was not an independent mortality predictor, fungal biofilm formation makes catheters important potential sources of candidemia and persistent fungemia. Their clinical effect should therefore be considered together with catheter-related infection, removal timing, source control, and overall disease severity.

The Pitt bacteremia score, originally developed for bacterial infections, has predicted mortality in Candida fungemia and *C. auris* candidemia.^4,19^ In our cohort, it was higher among non-survivors and prognostic in ROC analysis. Because Pitt and SOFA both assess acute illness severity, simultaneous inclusion could introduce collinearity; SOFA was selected for its broader assessment of organ dysfunction, and Pitt was evaluated separately.

Whether *C. auris* independently confers greater mortality than other yeast species remains uncertain: adjusted comparative studies have reported conflicting results, possibly because of differences in case mix, healthcare exposure, comorbidity, treatment access, and infection severity. Our study lacked a comparator and therefore could not assess this question.^12,20^ Prospective studies incorporating treatment and management strategies are needed.

Among evaluable patients, 76.1% had prior *C. auris* colonization, with a median colonization-to-candidemia interval of 19 days, supporting evidence that skin colonization commonly precedes candidemia.^21,22^ However, colonization alone may not fully explain progression to invasive infection. In our previous case-control study, colonization was associated with candidemia only in univariable analysis, suggesting that progression depends on additional patient- and treatment-related factors.^23^ TPN, surgery, sepsis, antifungal exposure, arterial catheters, CVCs, chronic kidney disease, and multisite colonization have similarly been reported as risk factors for progression from colonization to infection.^24^

In the present cohort, the intervals from detected colonization, hospital admission, and ICU admission to candidemia were significantly shorter among non-survivors than among survivors. The occurrence of candidemia earlier in the hospital and ICU course may reflect a more rapid clinical trajectory among patients with greater baseline vulnerability and acute illness severity, consistent with their higher Pitt bacteremia and SOFA scores. However, the colonization-to-candidemia interval was calculated from the first detection of colonization rather than the actual time of acquisition, and screening was epidemiologically targeted rather than performed systematically at fixed intervals. Thus, the observed difference may partly reflect variation in screening and detection timing. Available surrogate measures did not demonstrate a greater fungal burden among non-survivors, because the Candida colonization index and blood culture time to positivity were similar between the groups. Catheter-related candidemia was also similarly distributed, suggesting that the confirmed catheter source did not explain the shorter intervals. Nevertheless, because other candidemia sources and possible extra-bloodstream infection sites could not be reliably established, a contribution from source-related differences cannot be excluded. These findings should therefore be considered hypothesis-generating rather than evidence of a causal relationship.

The higher frequency of TPN use among survivors should not be interpreted as a protective effect. One possible explanation is that TPN-associated candidemia may more often involve a catheter-related and potentially removable source, allowing more effective source control. However, this hypothesis could not be confirmed because only 14 patients received TPN and the frequency of catheter-related candidemia was similar between survivors and non-survivors. Similarly, the longer duration of mechanical ventilation before candidemia among survivors may reflect their longer hospital and ICU exposure before candidemia, whereas non-survivors developed candidemia earlier in their clinical course and had greater acute illness severity at candidemia onset. These findings may therefore reflect differences in infection timing, source, and residual confounding rather than protective effects of TPN use or prolonged mechanical ventilation.

TTP may reflect microbial burden and severity. In 57 *C. auris* candidemia cases, median TTP was 16.5 h and each 1-h decrease was associated with 1.39-fold higher odds of death.^25^ In our cohort, TTP neither differed by survival nor discriminated mortality. Blood volume, bottle number, pre-culture antifungal therapy, fungemia magnitude, and laboratory procedures may limit comparability across centers.

In our study, valvular vegetation and endophthalmitis were detected in 3.6% and 1.9% of patients, respectively. In a previous study evaluating 46 cases, the rates of endocarditis and endophthalmitis were each reported as 2.2%.^16^ Because not all patients underwent systematic evaluation for complications, our rates should be interpreted with caution and may not fully reflect the true burden of these complications.

Resistance was 95.1% for fluconazole, 31.2% for amphotericin B, 1.3% for anidulafungin, and 1.3% for micafungin. Seven isolates had a caspofungin MIC at or above the CDC tentative threshold; however, caspofungin results were interpreted cautiously and were not used to define echinocandin-class resistance. Caspofungin susceptibility testing is affected by intermethod and interlaboratory variability. In addition, paradoxical growth at higher caspofungin concentrations—the Eagle effect—may complicate endpoint interpretation and result in apparent caspofungin resistance, particularly when anidulafungin and micafungin MICs remain low. ^26^ Published rates vary widely—77.4%–98.3%, 15.2%–86.4%, 0%–9%, and 0%–4.3%, respectively—according to region, clade, population, method, and threshold.^5,7,11,12,16,17,20^ The low resistance rates to anidulafungin and micafungin in our cohort are consistent with the continued role of echinocandins as key treatment options. In the absence of CLSI- or EUCAST-established species-specific clinical breakpoints, CDC tentative breakpoints were used. Nevertheless, the use of reference-laboratory and commercial broth microdilution methods during different study periods may have introduced intermethod variability.

In the international prospective study including Türkiye, resistance was 90.4% for fluconazole, 9.0% for caspofungin, 0.8% for micafungin, 2.6% for anidulafungin, and 62.7% for amphotericin B, with corresponding MIC₅₀/MIC₉₀ values reported.^17^ Differences may reflect clade distribution, antifungal use, outbreak epidemiology, and laboratory methods. ICU clusters and high fluconazole resistance at our center underscore the need for local surveillance and antifungal stewardship.

MDR and PDR rates were 28.6% and no isolate met the revised definition of PDR, compared with 58.4% and 0.8%, respectively, in Erdem et al.^17^ and 65.4% and 3.8% in a smaller Turkish study.^27^ Direct comparison is limited by differences in MDR/PDR definitions, the echinocandins included in class-level assessment, susceptibility methods, and interpretive thresholds.

Limitations include the retrospective, single-center design; missing clinical and laboratory data; non-standardized complication screening; incomplete susceptibility testing and colonization screening. Susceptibility testing was performed using reference-laboratory and commercial broth microdilution methods during different study periods, which may have introduced intermethod variability. In addition, caspofungin MIC results may have been affected by limited reproducibility and the Eagle effect; therefore, they were interpreted descriptively and excluded from echinocandin-class resistance and MDR/PDR classifications. Elevated caspofungin MICs were not confirmed by repeat reference broth microdilution, and FKS1 sequencing was not performed, limiting microbiological confirmation of these elevated results. Furthermore, follow-up blood cultures were not obtained at standardized intervals; therefore, the observed time to culture clearance may have been influenced by the timing and frequency of blood culture sampling. Available-case analysis avoided assumptions from imputation but reduced some analysis samples. Although the duration of hospital exposure during the current admission before candidemia was captured by the admission-to-candidemia interval, the cumulative duration of separate hospitalizations during the preceding three months was not available. Prior hospitalization was therefore recorded as a binary variable, which may not fully reflect cumulative healthcare exposure, comorbidity burden, or the intensity of medical interventions. Lack of genomic analysis prevented assessment of clonal relatedness and resistance mechanisms. Information regarding the anatomical sites and diagnostic findings of possible concurrent extra-bloodstream *C. auris* infections was unavailable.

Strengths include a relatively large cohort of culture-confirmed *C. auris* candidemia and integrated assessment of colonization, devices, clinical and laboratory findings, treatment, microbiological outcomes, resistance, and mortality. Variable-specific denominators and missing data were transparently reported. The mortality model showed acceptable discrimination (AUC, 0.728) and good calibration (Hosmer–Lemeshow *P* = 0.947), although internal assessment may be optimistic and requires external validation.

In conclusion, 28-day mortality was primarily associated with comorbidity burden and acute organ dysfunction at candidemia onset. CVC removal was more frequent among survivors but, as a post-baseline variable susceptible to early-mortality and survivor bias, its causal effect could not be determined. Similarity in other management variables may reflect consistent care, although limited power and missingness remain possible. Recognition of high-risk patients and local susceptibility patterns may support empirical treatment, targeted colonization screening, antifungal stewardship, and infection-control priorities.
